# Dual Aldosterone- and Cortisol-Secreting Adrenal Cortical Carcinoma: Pre- and Perioperative Evaluation and Management

**DOI:** 10.1210/jcemcr/luad073

**Published:** 2023-07-12

**Authors:** Rebecca Rosenberg, Christopher D Raeburn, Michael R Clay, Margaret E Wierman

**Affiliations:** Division of Endocrinology, Diabetes, and Metabolism, University of Colorado Anschutz Medical Campus, Aurora, CO, USA 80045; Department of Veterans Affairs, Rocky Mountain Regional Veterans Medical Center, Aurora, CO, USA 80045; Division of GI, Trauma and Endocrine Surgery, University of Colorado Anschutz Medical Campus, Aurora, CO, USA 80045; Division of Endocrinology, Diabetes, and Metabolism, University of Colorado Anschutz Medical Campus, Aurora, CO, USA 80045; Department of Pathology, University of Colorado School of Medicine, Aurora, CO, USA 80045; Division of Endocrinology, Diabetes, and Metabolism, University of Colorado Anschutz Medical Campus, Aurora, CO, USA 80045; Department of Veterans Affairs, Rocky Mountain Regional Veterans Medical Center, Aurora, CO, USA 80045

**Keywords:** adrenal cortical carcinoma, hypercortisolism, hyperaldosteronism, mitotane

## Abstract

Adrenal cortical carcinoma (ACC) is a rare cancer (1-2/million) that presents with hormone overproduction in 60% of cases. Presentation of ACC with multiple hormone syndromes from different adrenal zones is rare. We present a case of dual-secreting ACC with hyperaldosteronism and cortisol excess. The previously healthy patient was noted to have new-onset hypertension and hypokalemia during a primary care visit. On hormonal evaluation, he was found to have evidence of hyperaldosteronism and adrenocorticotropic hormone (ACTH)-independent cortisol excess. Imaging revealed a 2.7 × 3.1 × 3.5 cm left adrenal mass with indeterminant computed tomography characteristics. He underwent laparoscopic adrenalectomy and required glucocorticoid replacement for adrenal insufficiency postoperatively. Pathology revealed stage T2N0M0 ACC. His hypokalemia resolved and glucocorticoids were stopped within a month. This case stresses the importance of routine screening for cortisol excess in all adrenal masses detected on imaging. Avoidance of postoperative adrenal insufficiency in patients with cortisol excess without overt Cushing syndrome is paramount.

## Introduction

Representing 0.3% of adrenal tumors, adrenal cortical carcinoma (ACC) is a rare and understudied disease [1]. Approximately 60% of patients present with hormone excess syndromes [1]. Of patients presenting with hormonal syndromes, the majority present with cortisol excess, while a smaller number present with symptoms of hyperandrogenism, hyperaldosteronism, or with increased estradiol levels [2]. ACC presentation with a combination of hormonal excess syndromes from different adrenal zones is rare and described previously only in 4 case reports [3-6]. Patients presented either with biochemical evidence of combined aldosterone and cortisol hypersecretion [5, 6], aldosterone, cortisol, and estradiol excess [4], or aldosterone, cortisol, and androgen excess [3]. Given the rarity of co-secreting tumors, the impact on morbidity or mortality compared to single-hormone-secreting ACC is unknown. As the first-line treatment for ACC is adrenalectomy, the existence of dual- and triple-secreting ACC emphasizes the importance of preoperative testing to avoid peri- and postoperative complications as well as for long term management. We describe a case of dual-secreting ACC in a patient presenting with hyperaldosteronism and incidental but clinically relevant cortisol excess.

## Case Presentation

A 63-year-old man presented to primary care for an annual exam. His past medical history was significant for well-controlled hyperlipidemia on atorvastatin. Family history was negative for genetic syndromes that predispose to ACC. The patient was noted to have an elevated blood pressure of 148/92 mmHg with no prior hypertension. Other vital signs were normal and physical examination was normal. After lifestyle modification, blood pressure remained elevated at 172/98 mmHg.

## Diagnostic Assessment

**Figure 1.**
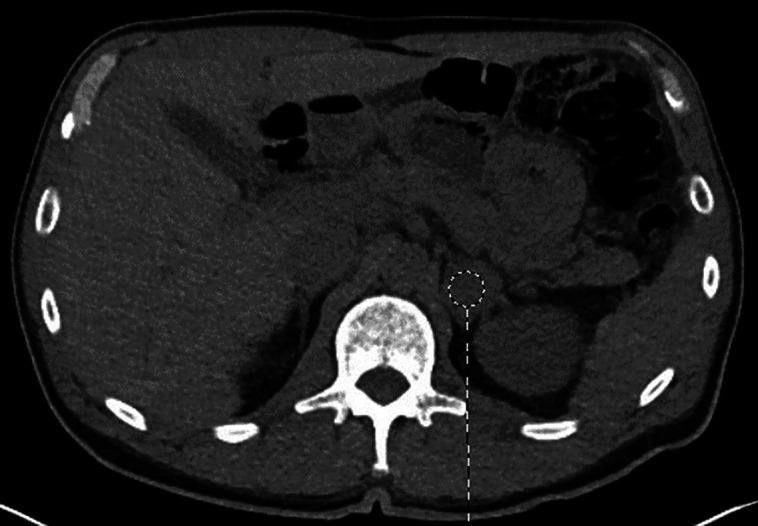
CT image of left adrenal mass (pre-contrast) of patient with aldosterone/cortisol dual-secreting ACC.

At follow-up with primary care, there were no symptoms of hypertensive urgency or cortisol excess aside from easy bruising. The physical examination demonstrated no abdominal striae, central obesity, or abnormal fat distribution. Antihypertensive medication (lisinopril-hydrochlorothiazide) was initiated. Basic metabolic panel was notable for a normal creatinine but hypokalemia with potassium level of 3.0 mmol/L (3.0 mEq/L; reference range, 3.5-5.1 mmol/L [3.5-5.1 mEq/L]). Following discovery of hypokalemia, hydrochlorothiazide was discontinued. Given the findings of hypertension and hypokalemia, evaluation for serum aldosterone level and plasma renin activity were obtained. Laboratory evaluation was concerning for primary hyperaldosteronism with high-normal aldosterone at 0.78 nmol/L (782.3 pmol/L) (reference range, 0.11-0.86 nmol/L [110.96-859.9 pmol/L]) and suppressed plasma renin activity 2.3 pmol/L/h (0.1 ng/mL/h) (reference range, 23.7-71.1 pmol/L/h [1.0-30.0 ng/mL/h]), notably drawn while the patient was taking lisinopril. Abdominal computed tomography (CT) scan was performed, revealing a heterogeneous 2.7 × 3.1 × 3.5 cm left adrenal mass with an unenhanced CT attenuation of 31 Hounsfield units, with absolute washout of 71% and relative washout of 49.7% (Fig. 1). The right adrenal gland was normal.

Further laboratory evaluation confirmed adrenocorticotropic hormone (ACTH)-independent (ACTH value 1.9 pg/mL [0.42 pmol/L]; reference range, 7.2-63.3 pg/mL [1.59-13.94 pmol/L]) cortisol excess with overnight 1 mg dexamethasone suppression test cortisol of 358.6 nmol/L (13 ug/dL) (reference range, <52.4 nmol/L [<1.9 ug/dL]). Dehydroepiandrosterone sulfate (DHEA-S) and plasma metanephrines were within the reference range on preoperative evaluation. No further hormone testing was performed. The patient was referred to endocrine surgery who elected not to perform confirmatory testing for hyperaldosteronism given high clinical suspicion of hyperaldosteronism with significant hypokalemia and hypertension and plan for adrenalectomy regardless of further testing based upon imaging characteristics.

## Treatment

Because of the dual hormone hypersecretion and suspicious imaging characteristics (larger tumor size, indeterminate Hounsfield units, and delayed washout), adrenalectomy was recommended. Despite some concern for malignancy, the endocrine surgeon felt that a laparoscopic approach was appropriate as the tumor was well circumscribed. Per the hospital protocol, glucocorticoids were not given preoperatively with the diagnosis of mild autonomous cortisol secretion (MACS). Adrenalectomy was performed without intraoperative evidence of extra-adrenal extension. Endocrinology was consulted postoperatively, and a cosyntropin stimulation test confirmed adrenal insufficiency. Results showed a baseline morning cortisol of 165 nmol/L (6 ug/dL, drawn at 09:00; reference range, 110-606 nmol/L [4-22 ug/dL]) with stimulation to 165.5 nmol/L at 30 minutes (6 ug/dL; ref range, >496 nmol/L [>18 ug/dL]). Stress dose glucocorticoids were administered, followed by a rapid taper to physiologic doses. The patient recovered well with an unremarkable postoperative clinical course. He experienced an immediate improvement in blood pressure and was discharged without antihypertensive therapy and prednisone 5 mg daily for ACTH deficiency.

**Figure 2.**
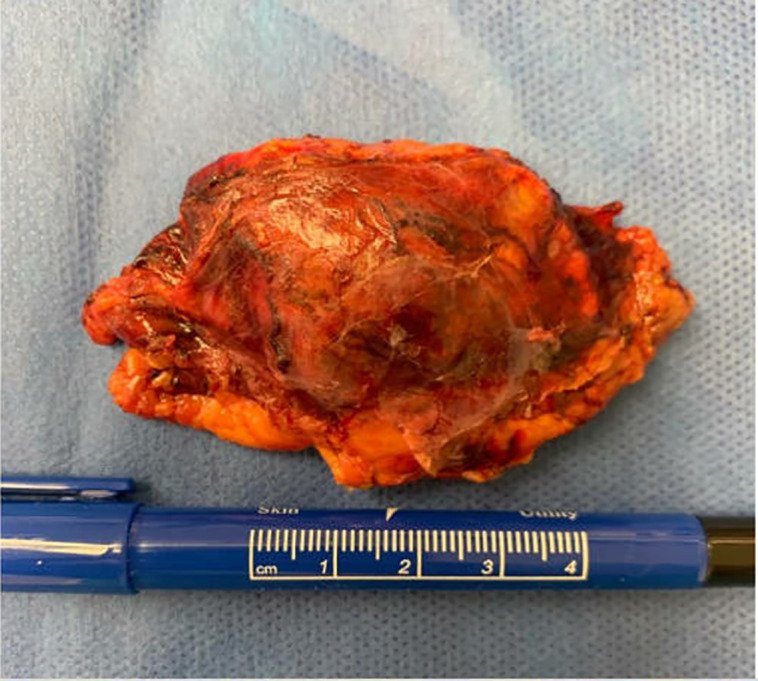
Macroscopic view of excised ACC.

**Figure 3.**
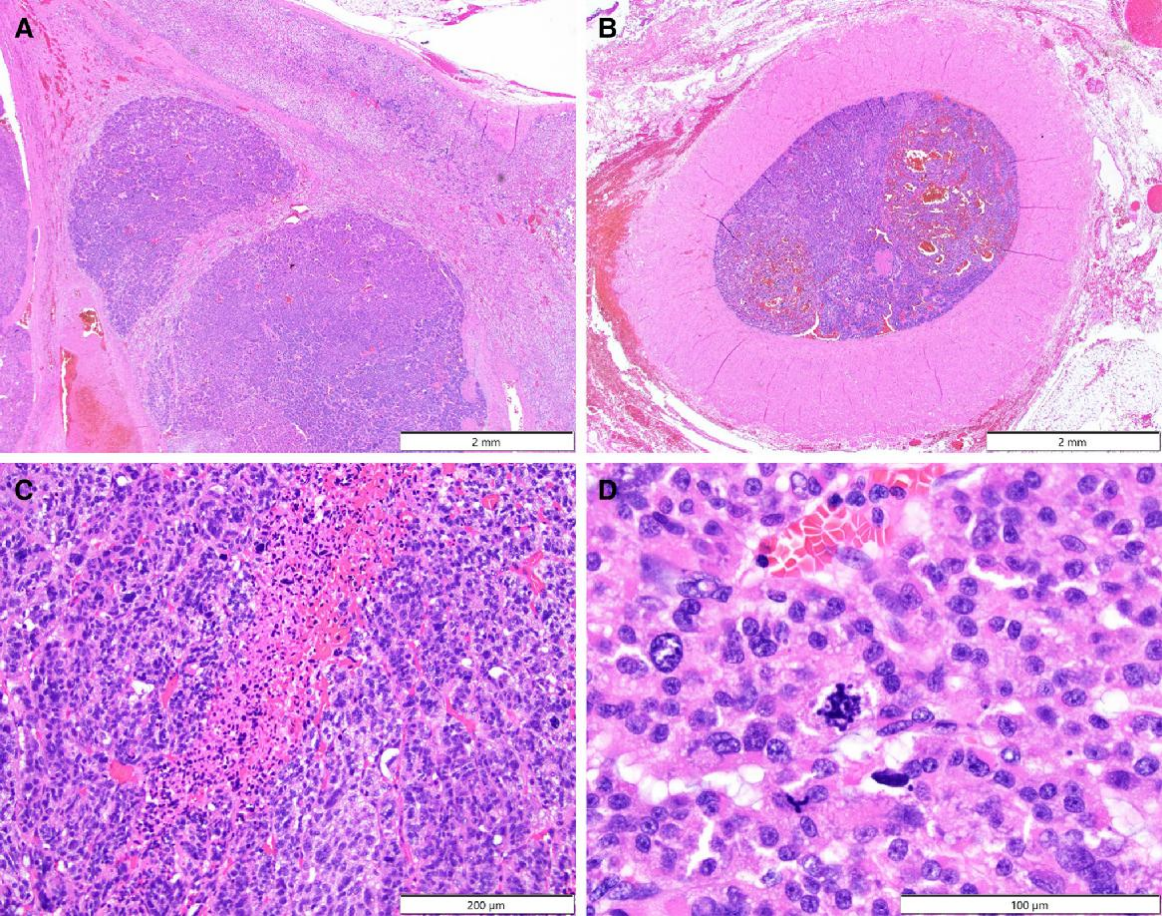
Histologic review of the adrenocortical mass shows close approximation with the native adrenal gland (A) with evidence of large vessel infiltration (B). Coagulative tumor necrosis (C) and atypical mitotic figures (D) were identified.

**Figure 4.**
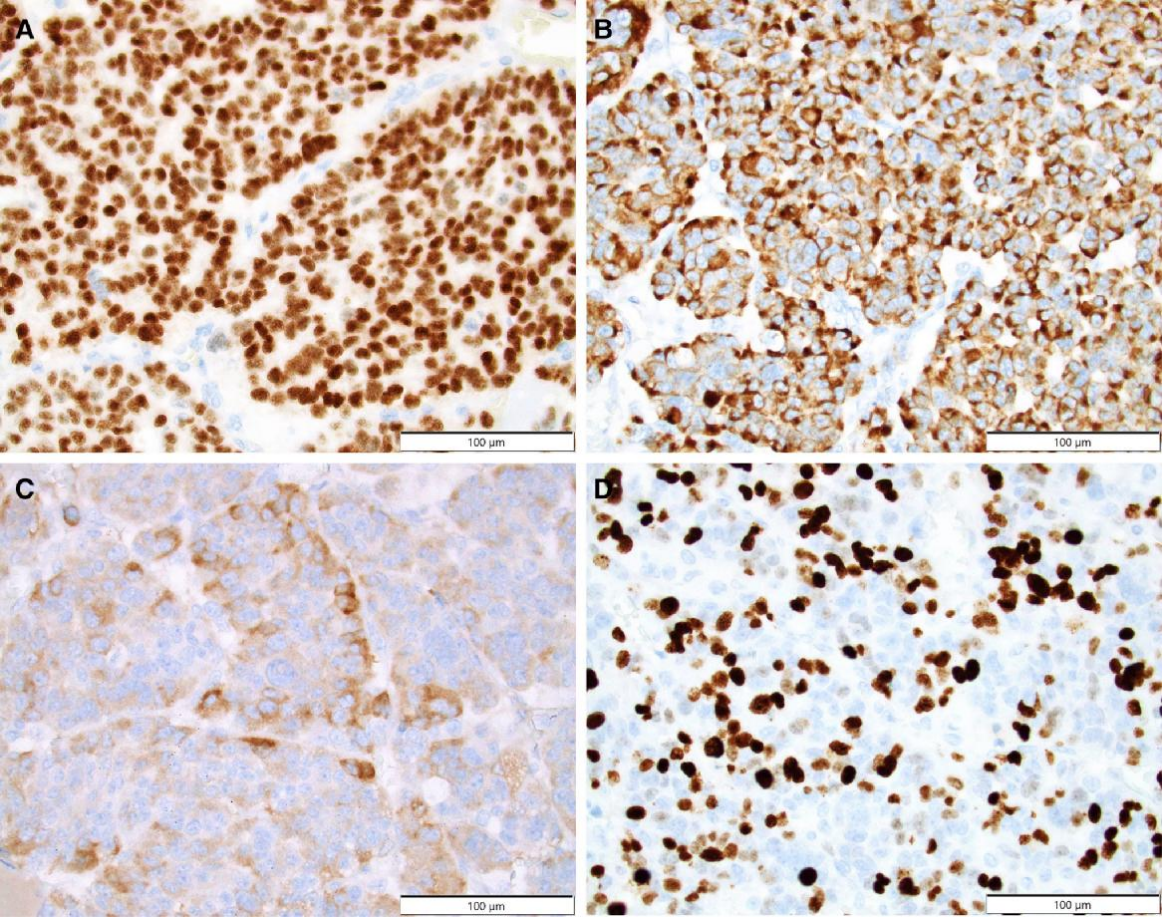
Immunohistochemical evaluation of the adrenocortical mass. Diffuse nuclear positivity for SF-1 staining (A) and cytoplasmic positivity for cytokeratin Cam 5.2 (B), supports the rendered diagnosis. Focal staining for Inhibin (C) was also identified. A Ki-67 stain (D) showed an elevated proliferative index.

Pathologic review of the gross specimen showed a sizable neoplasm measuring 6.5 × 3.0 × 2.5 cm (Fig. 2) which was larger when compared to the CT scan measurement performed approximately 6 weeks prior (2.7 × 3.1 × 3.5 cm). Histologic review demonstrated a high-grade ACC (Fig. 3A) with evidence of large vessel involvement (Fig. 3B). Coagulative tumor necrosis was present (Fig. 3C) as well as numerous atypical multipolar mitotic figures (68 mitoses per 50 high-power fields) (Fig. 3D). No capsular invasion was detected, and surgical margins were negative. Immunohistochemical staining was performed (Fig. 4) and showed positivity for SF1, cytokeratin Cam 5.2, and Inhibin, compatible with the rendered diagnosis. A Ki-67 stain showed a markedly elevated proliferation rate (68% per 50 high-power fields). The pathology report noted a modified Weiss score of 6, indicating high-risk of aggressive behavior [7]. Postoperatively, the patient received staging with a CT of the chest, abdomen, and pelvis which was negative other than a small liver lesion. This lesion was evaluated further with magnetic resonance imaging of the abdomen and percutaneous biopsy, demonstrating a benign hemangioma. The patient was therefore diagnosed with stage T2N0 ACC.

## Outcome and Follow-up

Postoperatively, the patient's blood pressure remained normal, and hypokalemia resolved without rebound hyperkalemia or decrease in renal function. Serum aldosterone normalized. There was recovery of the hypothalamic-pituitary-adrenal axis at 4 weeks, and steroid therapy was discontinued. Adjuvant mitotane therapy was initiated, given the apparent rapid growth of the tumor and presence of lymphovascular invasion. The patient was titrated to 1500 mg mitotane twice daily and ultimately required levothyroxine 125 mcg daily as well as prednisone 5 mg twice daily for mitotane-induced central hypothyroidism and adrenal insufficiency. His 3-month postoperative scan showed no evidence of disease. Because of the aggressive pathologic features and rapid growth, 4 cycles of adjuvant cisplatin/etoposide were recommended after multidisciplinary tumor board review. The patient remains disease free at 9 months.

## Discussion

The incidence of dual-, and even triple-, hormone-secreting ACC with a combination of aldosterone and steroid hormones is unknown, as only 4 case reports have been reported previously in the literature with varying presentations and clinical outcomes (Table 1) [3-6]. The mechanism of dual aldosterone/cortisol secretion is hypothesized to be due to overlap between genes coding for 11β-hydroxylase and aldosterone synthase, resulting in a chimeric gene with increased secretion of both enzymes, and, therefore, both hormones [8].

**Table 1.** Comparison of multiple-hormone-secreting ACCs in the literature

	Case study 1 [3]	Case study 2 [4]	Case study 3 [5]	Case study 4 [6]	Current case
**Patient age/sex**	59/F	19/M	26/F	37/F	63/M
**Presentation**	Hypertensive crisis, hypokalemia, hirsutism, weight gain, AUB	Leg weakness, hypertension (5 medications), hypokalemia	Arm weakness, hypertension, amenorrhea, hypokalemia	Leg weakness, fatigue, hypertension, facial plethora, hypokalemia	Incidental hypertension, hypokalemia
**Laboratory evaluation**	ARR 390 24h UFC 146 ug/24h ACTH <1 pg/mL Testosterone 229 ng/dL	ARR 1191.5 24h UFC 131.2 ug/24h	ARR >50 1 mg DST 27 mg/dL 8 mg DST 24 mg/dL 17(OH)P 14.5 ng/dL	ARR 39.8 24h UFC 1781.6 ug/24h Serum cortisol 42.26 ug/24h	ARR 282 ACTH 1.9 pg/mL 1 mg DST 13 ug/dL
**Radiographic evaluation**	CT: 2-cm right adrenal mass, no HU. Follow-up CT (1 year): 6.5-cm right adrenal mass, low washout	CT: 4.8-cm left adrenal mass, 26 HU	MRI: 8-cm left adrenal mass, no metastases	CT: 6-cm right adrenal mass, 2-cm paracaval LN	CT: 3.5-cm left adrenal mass, 31 HU
**Surgical management**	Adrenalectomy, no steroids	Laparoscopic adrenalectomy with steroids	Adrenalectomy with steroids	Open adrenalectomy	Laparoscopic adrenalectomy with steroids
**Medical management**	Mitotane	None	Steroid replacement	Adjuvant chemotherapy	Mitotane, adjuvant chemotherapy
**Pathology**	ACC, unknown features	ACC Ki67 index 5-10%	ACC + vascular and capsular invasion	Highly pleomorphic cells, anisonucleosis, high mitotic rate	ACC, Weiss score 6
**Outcome**	Metastases requiring chemotherapy and hepatic resection	All biochemical testing normal	Transient (∼1 y) AI	Liver metastases, hypokalemia, death at 11 months	Ongoing chemotherapy, no metastases

Abbreviations: 17(OH)P, 17α hydroxyprogesterone; AI, adrenal insufficiency; AUB, abnormal uterine bleeding; ARR, aldosterone to renin ratio; DST, dexamethasone suppression test; F, female; HU, Hounsfield units; LN, lymph node; M, male; UFC, urinary free cortisol.

Based on the available case reports, dual- or triple-secreting ACC cases present as a mix of both overt Cushing syndrome [3, 4, 6] and MACS (defined as morning cortisol after 1 mg dexamethasone suppression test greater than 1.8 µg/dL without overt signs of Cushing syndrome) [5]. No data are available as whether the presence of multiple hormonal secretion syndromes affects the likelihood of malignancy. Although both adrenal adenomas and ACC can present with hormone excess, there does not seem to be a difference in laboratory testing preoperatively in smaller lesions. The distinction in adenoma vs ACC is suggested from the adrenal CT scan. Radiographically, ACCs are typically >4 cm and with features of malignancy such as irregular borders, extra-adrenal extension, central necrosis, high attenuation (Hounsfield units >20), and/or delayed contrast washout (< 50%) [9]. The current case emphasizes that though these trends are true, the presence of a smaller adrenal mass on CT does not exclude ACC.

The current recommendations are to screen any adrenal mass for syndromes of biochemical excess: hyperaldosteronism (if hypertension and/or hypokalemia are present), hypercortisolism, and catecholamine excess [1]. Preoperative management of patients with hyperaldosteronism, either secondary to adrenal adenoma or ACC, focuses on maintenance of normal blood pressure, if possible, while avoiding exacerbation of hypokalemia [9]. Furthermore, the current Endocrine Society guidelines state that most patients with an abnormal aldosterone to renin ratio should undergo confirmatory testing; either with saline challenge, oral salt loading, captopril challenge, or fludrocortisone suppression [9]. However, in the setting of spontaneous hypokalemia, undetectable renin, and plasma aldosterone concentration >20 ng/dL (550 pmol/L), there may be no need for further confirmatory testing [9]. In the current case, confirmatory testing and adrenal vein sampling were not performed as the tumor had suspicious imaging characteristics and was also found to be co-secreting cortisol, therefore warranting excision. However, it is important to note that both aldosterone and renin evaluation are highly affected by medications, including angiotensin-converting enzyme inhibitors, and variable even among patients with confirmed hyperaldosteronism [9].

Postoperatively, patients with hyperaldosteronism from an adrenal adenoma or ACC are at higher risk for transient hyperkalemia and kidney injury [9], although neither occurred in the current patient case. To decrease the risk of these complications, careful titration of antihypertensives and postoperative cessation of mineralocorticoid receptor agonist therapy and potassium supplementation is recommended [9].

Although most adrenal adenomas present as biochemically silent or with a single hypersecretion syndrome, a meta-analysis by Spath and coworkers estimated that 27% of patients with hyperaldosteronism secondary to an adrenal adenoma have laboratory evidence of cortisol excess [8]. Expert discussion regarding preoperative screening for cortisol excess suggests that up to 50% of patients with adrenal incidentalomas exhibit laboratory evidence of MACS [1]. Therefore, evaluation for hypercortisolism preoperatively and monitoring for adrenal insufficiency postoperatively to preempt crisis with steroid therapy is necessary in all patients with adrenal lesions.

The risk for postoperative adrenal insufficiency has only recently been reported in patients with MACS who undergo adrenalectomy. Wang, et al reported that, in patients with MACS, the incidence of adrenal insufficiency was approximately 17% in the perioperative period, with all patients achieving hypothalamic-pituitary-adrenal axis recovery at 3 months [10]. The authors suggest that the risk of perioperative adrenal insufficiency is correlated to the cortisol level after 1 mg dexamethasone suppression test, with levels between 1.8 and 5 ug/dL having a lower risk than cortisol levels greater than 5 ug/dL [10].

This case report stresses the importance of clinical and hormonal screening in patients with an adrenal mass detected on imaging. A complete biochemical hormone evaluation should be performed regardless of the discovery of other hormone-secreting syndromes, ideally preoperatively with a multidisciplinary team including endocrinology. The medical team should continue to bear in mind that the existence of one hypersecretory syndrome does not rule out the presence of a second or third, and hormonal excess syndromes may affect patient outcomes peri- and postoperatively.

## Learning Points

- Evaluation of any adrenal mass should include laboratory testing for hypercortisolism to avoid perioperative complications from adrenal insufficiency.
- Hormonal secretion from an ACC or adrenal adenoma can be a combination of any of the more common secretory syndromes, and therefore, thorough hormone evaluation should be performed.
- The multidisciplinary team for the evaluation of any adrenal mass should include endocrinology throughout the surgical process to evaluate for any hormonal excess or deficiency.

## Contributors

All authors made individual contributions to authorship. R.R., M.W., C.R., and M.C.: diagnosis, inpatient management, and postoperative management of the patient. R.R.: Manuscript draft. M.W., C.R., and M.C.: clinical management of the patient, manuscript edits. All authors reviewed and approved the final draft.

## Data Availability

Data sharing is not applicable to this article as no datasets were generated or analyzed during the current study.

## Abbreviations

ACC: adrenal cortical carcinoma
ACTH: adrenocorticotropic hormone
CT: computed tomography
MACS: mild autonomous cortisol secretion.
